# Comparison of sexual function and quality of life after pelvic trauma with and without Angioembolization

**DOI:** 10.1186/s41038-015-0022-8

**Published:** 2015-11-16

**Authors:** Naeem Goussous, Mark D. Sawyer, Lisa-Ann Wuersmer, Marianne Huebner, Molly L. Osborn, Martin D. Zielinski

**Affiliations:** 1 Department of Surgery, Mayo Clinic, Rochester, MN USA; 2 Department of Physical Medicine and Rehabilitation, Rochester, MN USA; 3 Division of Biomedical Statistics & Informatics, Mayo Clinic, Rochester, MN USA; 4 Division of Trauma, Critical Care and General Surgery, Mary Brigh 2-818, Mayo Clinic, 1216 Second Street SW, Rochester, MN 55902 USA

**Keywords:** Pelvic fracture, Angioembolization, Sexual function, Quality of life

## Abstract

**Background:**

The aim is to study the safety of Angioembolization on long-term sexual function and quality of life.

**Methods:**

IRB approval was gained to review the prospectively collected trauma database as well as prospective questionnaires of patients at least 1 year out from pelvic fractures that occurred between 1996 and 2009. Surveys included the SF36v2, Female Sexual Function Index and the International Index of Erectile Function. Values for each domain were compared between patients treated with AE and 2:1 case-matched control patients as well as between the national norms. Values are presented as percentages or means with 95 % CI. *P* < 0.05 was considered statistically significant.

**Results:**

Thirty Seven cases and 74 matched controls were identified. 42 patients completed the survey. There were 13 cases (12 males), and 29 controls (22 males). There was a higher ISS (Injury Severity Score) (32 vs 27; *p* = 0.048) in the cases, but no difference in pelvic AIS (Abbreviated Injury Severity Score) (3 vs 3). Both groups scored similarly in the SF36 in all domains, but the entire cohort scored lower than the national norms in the physical functioning (41.9 (37.8–46.0) vs50), role physical (40.9 (36.2–45.7) vs50), body pain 43.8 (40.7–46.9) vs50), role emotional 46.3 (42.8–49.8) vs50), and physical composite score (42.1 (38.0–46.3) vs50). All domains of the sexual function in both questionnaires showed significant impairment in our cohort compared with norms. Male cases had similar scores to the controls.

**Conclusion:**

Pelvic fractures portend a worse long-term QOL and sexual function than the general population. AE, however, does not have an additive affect to these indices.

## Background

Disruption of the bony pelvis constitutes 4.8 % of all trauma admissions. The main cause of death in these patients is hemorrhage with a mortality rate as high as 42 % [1]. Patients with pelvic injuries tend to be hemodynamically stable at presentation and generally can be managed successfully by blood product resuscitation to correct hypotension, coagulopathy and acidosis as well as pelvic stabilization to decrease pelvic volume and exert a tamponade effect on the hematoma [2]. Those patients that do not respond to this treatment, from 1.9–3.0 %, generally have uncontrolled arterial bleeding and will benefit from angioembolization (AE) [3–5].

AE has been associated with severe complications, usually as a result of bilateral internal iliac artery embolization [6]. There are reports, however, of complications that may not be life threatening, but impede quality of life (QOL) in the long term such as impotence and urinary retention [7]. Poor sexual function after pelvic trauma is a major concern in patients who survive the initial injury. It has been shown that 35.9 % of men and 39.6 % of women will report some kind of sexual impairment [8]. Despite decreasing blood supply to the pelvis and perineum, bilateral internal iliac artery embolization has not been shown to adversely affect sexual function in males with pelvic trauma. Using non-validated metrics, men with pelvic fractures who required AE have been shown to have similar sexual impairment to those patients with pelvic fractures but did not require AE [9].

The published data about sexual function post AE in pelvic fracture patients is centered on men using non validated methods. Additionally, AE’s effect on female sexual function has not been described. We aim to evaluate the effect that AE in the setting of pelvic fracture has on long-term sexual function and QOL in both men and women using validated questionnaires.

## Methods

Institutional Review Board (IRB) was obtained to review retrospectively patients undergoing AE as a result of their pelvic fractures admitted to our Level I Trauma Center between 1996 and 2009. Data from our prospectively collected trauma database was utilized to create a case match cohort with similar pelvic fractures that did not undergo AE. The control cohort was matched by a 2:1 ratio age (+/- 5 years), gender, severity of pelvic fracture (Abbreviated Injury Severity Score (AIS) +/- 1), hemodynamic stability (systolic blood pressure < or ≥ 90 mmHg on admission), and the presence of a genitourinary injury. Patients less than one year out from their traumatic incident were excluded [10]. Subselective AE was defined as embolization of a branch of the aorta or internal iliac artery. The STROBE Statement (STrengthening the Reporting of OBservational studies in Epidemiology) and observational study checklist was used in the preparation of this manuscript.

### Questionnaires

Eligible patients were asked to fill out two of three surveys, dependent on gender: Short Form 36 Health Survey version 2 (SF36) [11], Female Sexual Function Index (FSFI) [12], and International Index of Erectile Function (IIEF) [13]. Male sexual function was evaluated by IIEF, a validated brief self-administered questionnaire. Fifteen specific questions covering five relevant aspects of male sexual function (erectile function, sexual desire, orgasmic function, intercourse satisfaction and overall satisfaction) within the past 4 weeks were asked. Female sexual function was assessed using the 19-question instrument FSFI. This validated questionnaire evaluates six different aspects of female sexual function (desire, arousal, lubrication, orgasm, satisfaction, pain). QOL was evaluated using the SF36. This questionnaire has been validated to assess the patients’ mental and physical well being and the impact of different health conditions on patients’ QOL. This questionnaire consists of 36 questions and it assesses health status in 8 different domains: physical function (PF), role physical (RP), body pain (BP), general health (GH), vitality (Vi), social function (SF), role emotional (RE), mental health (MH). Questions are combined to form 2 composite scores: the physical composite score (PCS) and the mental composite score (MCS). The two questions looked into the presence or absence of urinary incontinence and incomplete bladder evacuation. The Mayo Clinic Survey Research Center was responsible for administering the survey. The survey booklet was sent twice, one month apart. If no reply was received, a telephone call was made to remind potential subjects of their opportunity to participate. No further contact was made.

### Statistics

Continuous variables are presented as medians and interquartile range (IQR). Categorical variables are presented as percentages. Wilcoxon Two-Sample Test was used to compare the results of the continuous variables between the 2 groups. And for the categorical variables, Fisher’s Exact Test was used. The score results of the surveys were compared between both groups using Wilcoxon Two – Sample Test. Survey results are presented as means and standard deviations. Statistical significance was defined as a p value <0.05 for all comparisons.

The whole cohort results in the survey were compared to norms. Results are presented as means and 95 % confidence interval. For the SF36 we used US national norms and for the IIEF and FSFI we used means of healthy controls identified by Rosen et al. [12, 13] We considered our group not different from these values if the confidence limits included the control mean.

## Results

Overall, 2056 patients were identified with pelvic fractures over the study period. Of these, 37 underwent AE. These patients were case matched to 74 control patients (Table 1). Therefore, the total patient population that received surveys was 111 (81 males and 30 females). These patients presented with a median (IQR) age of 49 (35–72), median ISS (Injury Severity Score) of 25 (19–34) and a median pelvic AIS of 3 (2–4). All patients had blunt trauma as their mechanism of injury. The mean time from injury to enrolment was 6 years.

**Table 1:** Survey completion rates by gender of the 111 patients eligible to enroll

Feature	Overall (*n* =111)	Men (*n* = 81)	Women (*n* = 30)
Angioembolization	37	27	10
Angioembolization patients completing survey	13 (35 %)	12 (44 %)	1 (10 %)
Case matched controls	74	54	20
Controls completing survey	29 (39 %)	22 (41 %)	7 (35 %)

Overall, 42 patients successfully completed the survey (38 %) and 33 (30 %) did not. Of the remaining 36 potential subjects, 20 refused enrollment, 8 could not be reached due to a lack of a telephone number, 5 could not complete the survey (2 secondary to a language barrier), 2 returned a blank survey and 1 died.

Of the 42 patients that were enrolled, 13 underwent angioembolization with 29 controls. The average age of responders was 56 years with 81 % male. There were multiple types of pelvic fractures: 33 % pubic rami, 26 % acetabular, 29 % sacral/ coccygeal, and 17 % iliac. Two patients had open fractures. Four patients from the AE group had bilateral internal iliac embolization, 5 had unilateral embolization of an internal iliac artery, 2 patients had unilateral embolization of the superior gluteal artery, 1 had the inferior epigastric embolized, and the last had embolization of 3 lumbar arteries. 62 % of the embolizations were subselective. Patients in the AE group had higher ISS and a lower TRISS (Trauma and Injury Severity Score) scores, but a similar pelvic AIS compared to the control group (Table 2).

**Table 2:** Demographics and clinical data comparing AE to controls in patients who returned the survey

Feature	Control (*n* = 29)	AE (*n* = 13)	*P* Value
Age	51 (39–71)	67 (47–78)	0.161
Male	75.9 %	92.3 %	0.398
Diabetes	3.5 %	7.7 %	0.529
Hypertension	20.7 %	7.7 %	0.405
Renal Disease	3.5 %	0.0 %	1.0
Hyperlipidemia	10.7 %	0.0 %	0.539
Smoking	27.6 %	30.8 %	1.0
ED heart rate	98 (87–117)	101 (70–117)	0.697
ED systolic blood pressure	130 (109–152)	114 (92–132)	0.175
ISS	27 (15–29)	34 (22–39)	0.048
TRISS	0.97 (0.95–0.98)	0.89 (0.70–0.97)	0.019
Pelvic AIS	3 (3–4)	3 (2–5)	0.356
ED GCS	15 (15–15)	15 (8–15)	0.795
ED trauma score	12 (12–12)	12 (11–12)	0.611
Calculated RTS	7.8 (7.8–7.8)	7.8 (7.3–7.8)	0.058
Hospital length of stay (Days)	16 (8–26)	17 (15–77)	0.105

Continuous data are presented as median (IQR)

*ISS* injury severity score, *TRISS* trauma injury severity score, *AIS* abbreviated injury score, *GCS* glasgow coma scale, *ED* emergency department, *RTS* revised trauma score, *IQR* interquartile range

AE and control groups had similar scores in all the domains of the SF36 questionnaire (Table 3). Comparison of both trauma cohorts to the national normative values demonstrates a significant decline in the QOL in the trauma patients. Specifically, these patients were worse off in the PF, RP, BP, RE domains and the PCS (Table 4).

**Table 3:** Results of the Short Form 36 Health Survey in patients undergoing angioembolization compared to control

Feature	Control (*n* = 29)	AE Group (*n* =13)	P
Physical Functioning (PF)	41.1 (+/- 13.8)	43.6 (+/- 11.6)	0.71
Role Physical (RP)	40.0 (+/- 15.4)	43.0 (+/- 14.4)	0.60
Body Pain (BP)	42.4 (+/- 10.2)	46.8 (+/- 8.9)	0.24
General Health (GH)	47.7 (+/- 13.2)	52.0 (+/- 8.0)	0.62
Vitality (Vi)	48.3 (+/- 11.0)	51.2 (+/- 9.1)	0.30
Social Function (SF)	47.4 (+/- 11.6)	47.8 (+/- 11.3)	0.98
Role Emotional (RE)	45.1 (+/- 11.7)	48.8 (+/- 9.6)	0.42
Mental Health (MH)	48.0 (+/- 10.8)	52.9 (+/- 6.6)	0.19
Mental Composite Score (MCS)	49.3 (+/- 11.6)	52.8 (+/- 8.8)	0.48
Physical Composite Score (PCS)	41.4 (+/- 14.3)	43.7 (+/- 10.4)	0.81

Results are presented as means (standard deviations)

**Table 4:** Comparison between the scores for the various domains of the Short Form 36 version 2 questionnaire and US national norms

Feature	Cohort (*n* = 42)	Norms	Difference (Higher/Within/Lower)
Physical Functioning (PF)	41.9 (37.8–46.0)	50	Lower
Role Physical (RP)	40.9 (36.2–45.7)	50	Lower
Body Pain (BP)	43.8 (40.7–46.9)	50	Lower
General Health (GH)	49.0 (45.3–52.7)	50	Within
Vitality (Vi)	49.2 (46.0–52.5)	50	Within
Social Function (SF)	47.5 (44.1–51.1)	50	Within
Role Emotional (RE)	46.3 (42.8–49.8)	50	Lower
Mental Health (MH)	49.5 (46.4–52.6)	50	Within
Mental Composite Score (MCS)	50.4 (47.0–53.8)	50	Within
Physical Composite Score (PCS)	42.1 (38.0–46.3)	50	Lower

Data are presented as means with 95 % confidence intervals in parentheses. Difference is assumed if control falls outside the 95 % confidence interval of the cohort

The results of the IIEF questionnaire were similar in all domains for the AE and control groups (Table 5). There were significant detriments to male sexual function inclusive of both trauma cohorts compared to normative controls (Table 6) [13]. Fourteen trauma patients reported no sexual activity in the 4 weeks prior to the survey.

**Table 5:** Comparison of International Index of Erectile Function (IIEF) domains in men

Domain	Control (*n* = 22)	AE Group (*n* = 12)	*P* Value
Erectile function	14.4 (+/- 11.6)	7.0 (+/- 10.3)	0.075
Orgasmic function	5.3 (+/- 4.6)	2.5 (+/- 3.8)	0.115
Sexual desire	5.5 (+/- 2.5)	4.9 (+/- 2.0)	0.507
Intercourse satisfaction	5.8 (+/- 5.6)	2.6 (+/- 5.3)	0.162
Overall satisfaction	5.7 (+/- 2.7)	5.8 (+/- 3.0)	0.941

Data are presented in means with standard deviation in parentheses

**Table 6:** Comparison of the International Index of Erectile Function (IIEF) domains and the Female Sexual Function Index (FSFI) domains to Rosen’s controls

IIEF domains	Cohort (*n* = 34)	Rosen’s controls	FSFI domains	Cohort (*n* = 8)	Rosen’s controls
Erectile function*	11.9 (7.7–16.0)	25.8	Desire*	2.4 (1.0–3.8)	4.1
Orgasmic function*	4.3 (2.7–6.0)	8.8	Arousal*	1.8 (0.0–3.6)	5.0
Sexual desire*	5.3 (4.5–6.2)	7.0	Lubrication*	1.8 (0.0–3.7)	5.6
Intercourse satisfaction*	4.7 (2.7–6.7)	10.6	Orgasm*	2.2 (0.0–4.4)	5.1
Overall satisfaction*	5.7 (4.7–6.8)	8.6	Satisfaction	3.6 (2.0–5.2)	5.1
			Pain*	2.1 (0.0–4.5)	5.6
			Full score*	13.9 (3.4–24.2)	30.5

Data are presented as means with 95 % confidence intervals in parentheses and compared to Rosen’s controls. *Signifies lower results than controls [12, 13]

There were 4 male patients who underwent AE of the internal iliac artery bilaterally. No significant differences in the results of the different domains were found between these 4 patients and the rest of the cohort who underwent AE (Table 7).

**Table 7:** Comparison of International Index of Erectile Function (IIEF) domains between patients who had bilateral AE of the internal iliac to those who did not

Domain	Bilateral internal iliac AE (*n* = 4)	Non bilateral internal iliac AE (*n* = 8)	*P* Value
Erectile function	10.3 (+/-13.2)	4.9 (+/-8.4)	0.40
Orgasmic function	2.5 (+/- 5.0)	2.1 (+/-3.3)	0.88
Sexual desire	5.5 (+/-1.7)	4.0(+/-2.6)	0.33
Intercourse satisfaction	3.8 (+/-7.5)	1.6 (3.9)	0.52
Overall satisfaction	6.0 (+/- 2.9)	3.8 (+/-3.6)	0.31

Data are presented in means with standard deviation in parentheses

Of the 10 female patients who were treated by AE, one patient returned the survey. This survey responder had no sexual activity in the 4 weeks prior to enrollment. Therefore, no comparison between females undergoing AE versus controls could be performed. Seven women from the control cohort returned the survey. These patients were compared to norms with Rosen’s controls (Table 6) [12]. The females in our cohort scored lower in all domains of the FSFI except for the satisfaction domain in which the mean of Rosen’s controls fell within the 95 % confidence interval of our cohort. Out of the 8 patients who returned the survey only four reported sexual activity in the 4 weeks prior to the survey.

## Discussion

Sexual dysfunction after major trauma is a significant long term problem in those patients that survive their injuries. The causes for this are multifactorial [14]. Magnetic resonance imaging and duplex ultrasound results show that there is an underlying vascular cause to impotence after pelvic trauma in up to 80 % of affected men [15]. Conversely, Mark et al. suggested a neurogenic cause for impotence; this was evident by a successful response to self injection of vasoactive agents. They concluded that bilateral pubic rami fractures carry a high risk for impotence due to disruption of the cavernosal nerves [16]. Decreased blood flow to the penis has been observed in renal transplant patients where the donor’s renal artery has been anastomosed in an end to end fashion to the recipient’s internal iliac artery, but in the absence of atherosclerosis no negative effects on erectile function has been observed [17]. In females, damage of the soft tissue in the perineum with resultant scar tissue formation and the residual deformity of the posterior pelvic ring and its associated posttraumatic arthritis of the sacroiliac joints among other have been considered as plausible causes for dyspareunia [18, 19]. Psychogenic factors manifested by anxiety, depression and post traumatic stress also have an adverse effect on sexual life [20].

The use of AE in hemorrhaging vessels is an essential modality for localizing and treating arterial bleeding in patients presenting with pelvic fractures and hemodynamic compromise. This modality has been associated with a lower blood product transfusion requirement and is generally considered an appropriate replacement to operative management [21]. The relative safety of AE in the short term has been demonstrated. In a prospective study, Velmahos et al. stated that AE should be used “liberally” in the setting of pelvic fractures complicated by hemorrhage [22]. There are reports, however, of short and long term complications such as perineal necrosis as well as buttock, thigh and perineal paresthesia, particularly after undergoing bilateral internal iliac artery AE [7]. There is limited data, however, on the effect that these complications have on long term sexual function, particularly in women.

The FSFI and the IIEF are validated questionnaires that provide quantitative measures assessing various aspects of male and female sexual function. Using the IIEF questionnaire for men, Malavaud et al. found a 30 % incidence of erectile dysfunction in patients sustaining pelvic fractures. Their patient group scored significantly lower in the overall satisfaction domain than controls [23]. Metze et al. reported short term sexual impairment as high as 61 % in men after pelvic injury. Over half of their cohort had erectile dysfunction post injury with the majority recovered within 12 months [10]. In women, many reports found that pelvic fractures had been associated with sexual impairment, dyspareunia and difficulty with vaginal delivery [24]. In a study by McCarthey et al. studying sexual function and QOL in female patients post pelvic fractures, they found that 51 % of the patients reported feeling less sexual pleasure and 19 % reported dyspareunia. The QOL of their cohort was significantly lower than normal controls as evidenced by the low scores in almost all the domains of the SF36 questionnaire [25]. Black et al. reported a 61 % incidence of sexual dysfunction in 13 female patients following pelvic fracture associated with urethral and bladder neck injury [26]. In the only study studying sexual function after AE, Ramirez et al. considered the effect that bilateral internal iliac AE had on male sexual function after pelvic fractures. In their report, they compared patients who had AE to two matched groups; 1) patients with pelvic fractures that did not require AE and 2) a control group of trauma patients without pelvic injury. Both pelvic fracture groups showed significantly compromised sexual function compared to the group without pelvic fractures and concluded that male sexual dysfunction was due to the pelvic fracture, not AE [9]. Non-validated questions were used, however, to reach these conclusions.

Our results confirm these findings; AE does not contribute to poor long term sexual function over and above the dysfunction suffered from pelvic injuries. Within our total population, there were equivalent rates of sexual dysfunction, as based on the IIEF and the FSFI results, regardless of whether or not AE was performed.

Disappointingly, only 1 woman that underwent AE completed the survey. After combining all female subjects (i.e. with and without AE) completing the FSFI, we demonstrated poorer overall long term sexual function compared to the normal control population. For men, while there was a significant reduction in long term sexual function compared to normal controls, AE did not play a role in this decline. The possibility for type II error exists in the erectile function domain, however. Those patients that underwent AE had a score less than half of those subjects that did not undergo AE. This *p* value was close to the 0.05 considered significant. A possible cause for this result may lie within the age of our cohort. Our overall group was older than other reported populations. There is a possibility that the reduction in blood flow that results in AE impacts elderly men more significantly than younger patients.

QOL results, as judged by the SF36, demonstrated similar findings to long term sexual function; AE itself is not the cause poor QOL, but rather, pelvic trauma is. Those patients with pelvic trauma had similar scores regardless of the need for AE. When comparing all pelvic fracture patients to normal controls, there were significantly lower scores in 4 of the 8 domains.

Our study has some limitations. First, despite that the response rate for our survey is on par with other questionnaire studies, a 39 % survey completion rate limited our conclusion. Not only are trauma patients notorious for poor follow-up rates, we believe that the topic of the questionnaire significantly impacted the return rate. Due to the design of our study, we were able to call potential subjects. A significant number of patients, particularly women, refused to complete the survey due to the nature of the questions asked. Sexual function is a difficult topic for any person to consider, particularly after the severe body image perceptions that can result from trauma. Both sexual function surveys ask graphic questions related to lubrication, dyspareunia and orgasm, information some potential subjects were unwilling to share. As a result, we were unable to complete one of our aims; that of evaluating long term sexual function in women after AE. In addition to preventing us from performing one of our stated aims, the lack of data from 61 % of potential subjects biased the results and may have resulted in further type II error given the small number of responders. Second, there is an average of 6 years delay between the onset of injury and the time of survey enrolment, this could have added an age related decline in sexual function. Third, this is a retrospective study, making it hard to draw a cause and effect relationship.

## Conclusion

AE did not have an additional adverse impact to sexual function and QOL to the injuries sustained by pelvic trauma. AE should be used when indicated to control hemorrhaging vessels of the pelvis without concern for detrimental long term effects.

## References

[1] Mucha P, Farnell MB (1984). Analysis of pelvic fracture management. J Trauma.

[2] Cullinane DC, Schiller HJ, Zielinski MD, Bilaniuk JW, Collier BR, Como J (2011). Eastern association for the surgery of trauma practice management guidelines for hemorrhage in pelvic fracture-update and systematic review. J Trauma.

[3] Mucha P, Welch TJ (1988). Hemorrhage in major pelvic fractures. Surg Clin North Am.

[4] Angolini SF, Shah K, Jaffe J, Newcomb J, Rhodes M, Reed JF (1997). Arterial embolization is a rapid and effective technique for controlling pelvic hemorrhage. J Trauma.

[5] Velmahos GC, Chawan S, Hanks SE, Murray JA, Berne TV, Asensio J (2000). Angiographic embolization of bilateral internal iliac arteries to control life threatening hemorrhage following blunt trauma to the pelvis. Am Surg.

[6] Takahira N, Shindo M, Tanaka K, Nishimaki H, Ohwada T, Itoman M (2001). Gluteal muscle necrosis following transcatheter angiographic embolization for retroperitoneal hemorrhage associated with pelvic fracture. Injury.

[7] Ben-Menachem Y, Coldwell DM, Young JW, Burgess AR (1991). Hemorrhage associated with pelvic fractures: causes, diagnosis, and emergency management. Am J Radiol.

[8] Harvey-Kelly KF, Kanakaris NK, Eardley I, Giannoudis PV (2011). Sexual function impairment after high energy pelvic fractures: evidence today. The Journel of Urology.

[9] Ramirez JI, Velmahos GC, Best CR, Chan LS, Demetriades D (2004). Male sexual function after bilateral internal iliac artery embolization for pelvic fracture. J Trauma.

[10] Metze M, Tiemann AH, Josten C (2007). Male sexual dysfunction after pelvic fracture. J Trauma.

[11] Ware JE, Gandek B (1998). Overview of the SF-36 health survey and the international quality of life assessment (IQOLA) project. J Clin Epidemiol.

[12] Rosen R, Brown C, Heiman J, Leiblum S, Meston C, Shabsigh R (2000). The Female Sexual Function Index (FSFI): a multidimensional self-report instrument for the assessment of female sexual function. J Sex Marital Therapy.

[13] Rosen R, Riley A, Wagner G, Osterloh I, Kirkpatrick J, Mishra A (1997). The International Index of Erectile Function (IIEF): a multidimensional Scale for the Assessment of Erectile Dysfunction. Urology.

[14] Sorensen MD, Wessells H, Rivara FP, Zonies DH, Jurkovich GJ, Wang J (2008). Prevalence and predictors of sexual dysfunction 12 months after major trauma: a national study. J Trauma.

[15] Armenakas NA, McAninch JW, Lue TF, Dixon CM, Hirack H (1993). Posttraumatic impotence: magnetic resonance imaging and duplex ultrasound in diagnosis and management. J Urol.

[16] Mark SD, Keane TE, Vandermark RM, Webster GD (1995). Impotence following pelvic fracture urethral injury: incidence, aetiology and management. Br J Urol.

[17] El-Bahnasawy MS, El-Assmy A, Dawood A, Abobieh E, Dein BA, El-Din AB (2004). Effects of the use of internal iliac artery for renal transplantation on penile vascularity and erectile function: a prospective study. J Urol.

[18] Vallier HA, Cureton BA, Schubeck D (2021). Pelvic ring injury is associated with sexual dysfunction in women. J Orthop Trauma.

[19] Copeland CE, Bosse MJ, McCarthy ML, Mackenzie EJ, Guzinski GM, Hash CS (1997). Effect of trauma and pelvic fracture on female genitourinary, sexual, and reproductive function. J Orthop Trauma.

[20] Kotler M, Cohen H, Aizenberg D, Matar M, Loewenthal U, Kaplan Z (2000). Sexual dysfunction in male posttraumatic stress disorder patients. Psychother Psychosom.

[21] Panetta T, Scalfani SJ, Goldstein AS, Phillips TF, Shaftan GW (1985). Percutaneous transcatheter embolization for massive bleeding from pelvic fractures. J Trauma.

[22] Velmahos GV, Toutouzas KG, Vassiliu P, Sarkisyan G, Chan LS, Hanks SH (2002). A prospective study on the safety and efficacy of angiographic embolization for pelvic and visceral injuries. J Trauma.

[23] Malavaud B, Mouzin M, Tricoire JL, Game X, Rischmann P, Sarramon JP (2000). Evaluation of male sexual function after pelvic trauma by the international index of erectile function. Urology.

[24] Canada LK, Barr J (2010). Pelvic fractures in women of child bearing age. Clin Orthop Relat Res.

[25] McCarthay ML, Mackenzie EJ, Bosse MJ, Copeland CE, Hash CS, Burgess AR (1995). Functional status following orthopedic trauma in young patients. J Trauma.

[26] Black PC, Miller EA, Porter JR, Wessells H (2006). Uretheral and bladder neck injury associated with pelvic fracture in 25 female patients. J Urol.

